# Collagen Fragments Produced in Cancer Mediate T Cell Suppression Through Leukocyte-Associated Immunoglobulin-Like Receptor 1

**DOI:** 10.3389/fimmu.2021.733561

**Published:** 2021-10-07

**Authors:** Saskia V. Vijver, Akashdip Singh, Eline T. A. M. Mommers-Elshof, Jan Meeldijk, Ronald Copeland, Louis Boon, Sol Langermann, Dallas Flies, Linde Meyaard, M. Inês Pascoal Ramos

**Affiliations:** ^1^ Center for Translational Immunology, University Medical Center Utrecht, Utrecht University, Utrecht, Netherlands; ^2^ Oncode Institute, Utrecht, Netherlands; ^3^ NextCure, Beltsville, MD, United States; ^4^ Polpharma Biologics, Utrecht, Netherlands

**Keywords:** LAIR-1, collagen, ECM - extracellular matrix, T cells, cancer, immunotherapy

## Abstract

The tumor microenvironment (TME) is a complex structure comprised of tumor, immune and stromal cells, vasculature, and extracellular matrix (ECM). During tumor development, ECM homeostasis is dysregulated. Collagen remodeling by matrix metalloproteinases (MMPs) generates specific collagen fragments, that can be detected in the circulation of cancer patients and correlate with poor disease outcome. Leukocyte-Associated Immunoglobulin-like Receptor-1 (LAIR-1) is an inhibitory collagen receptor expressed on immune cells in the TME and in the circulation. We hypothesized that in addition to ECM collagen, collagen fragments produced in cancer can mediate T cell immunosuppression through LAIR-1. Our analyses of TCGA datasets show that cancer patients with high tumor mRNA expression of MMPs, collagen I and LAIR-1 have worse overall survival. We show that *in vitro* generated MMP1 or MMP9 collagen I fragments bind to and trigger LAIR-1. Importantly, LAIR-1 triggering by collagen I fragments inhibits CD3 signaling and IFN-γ secretion in a T cell line. LAIR-2 is a soluble homologue of LAIR-1 with higher affinity for collagen and thereby acts as a decoy receptor. Fc fusion proteins of LAIR-2 have potential as cancer immunotherapeutic agents and are currently being tested in clinical trials. We demonstrate that collagen fragment-induced inhibition of T cell function could be reversed by LAIR-2 fusion proteins. Overall, we show that collagen fragments produced in cancer can mediate T cell suppression through LAIR-1, potentially contributing to systemic immune suppression. Blocking the interaction of LAIR-1 with collagen fragments could be an added benefit of LAIR-1-directed immunotherapy.

## Introduction

The tumor microenvironment (TME) is immunosuppressive and can mediate resistance to therapies, including current immunotherapies (1). The TME consists of different cell types - such as tumor cells, cancer-associated fibroblasts (CAFs), and multiple types of immune cells - and the extracellular matrix (ECM) (2). Changes in the ECM have been linked to poor prognosis in cancer patients (3). These changes can be grouped into four remodeling processes. These include ECM deposition, which modifies the abundance and composition of ECM (4) and ECM post-translational modifications, altering the structural and biochemical characteristics of the ECM (5). Furthermore, ECM proteolytic degradation results in the release of ECM-bound factors (6) and bioactive ECM fragments (7) that can regulate cellular activation and migration. Lastly, force-mediated physical remodeling impacts ECM organization by increasing stiffness (8) and alignment of ECM fibers (9, 10).

Collagen is the most abundant component of the ECM, contributing to the stability and integrity of tissues (11). In the TME, collagen is produced by tumor cells (12), tumor-associated macrophages (TAMs) (12) and CAFs (13). The collagen structure in the ECM of the TME is stiffer and more linearized compared to healthy ECM (14). In many types of tumors increased collagen expression, density and alignment have been associated with poor prognosis (3).

The balance between matrix production and degradation is tightly regulated by, among others, the actions of matrix metalloproteinases (MMPs) and tissue inhibitors of MMPs (TIMPs). MMPs are zinc-dependent endopeptidases that degrade ECM components, including collagen (15). Many MMPs are upregulated in cancer, although their expression differs per tumor type (16). Tumor cells (2, 16), TAMs (2), CAFs (2, 17) and tumor-associated neutrophils (TANs) (18) produce MMPs in the TME. MMPs cleave multiple types of collagen into specific fragments. Several fragments have been identified in the serum of cancer patients, such as C1M. This collagen fragment, derived from type I collagen (19), is increased in the serum of cancer patients, and is associated with poor prognosis (20, 21). Additionally, high levels of collagen fragments, among which C1M, associate with a poor response to anti-CTLA-4 therapy in metastatic melanoma patients (22).

Checkpoint inhibition therapy targets immune inhibitory receptors, such as CTLA-4 and PD-1 (23). Leukocyte-Associated Immunoglobulin-like Receptor 1 (LAIR-1), also called CD305, is an inhibitory receptor present on almost all immune cell populations (24). LAIR-1 has been shown to inhibit immune cell functions of many immune cells present in the TME, including T cells (25). LAIR-1 expressing T cells are important players in the anti-tumor immune response (26), and dysfunction of T cells by signals from the TME hampers the anti-tumor activity (27). Collagens (28), including tumoral collagen (29), and proteins with collagen-like domains, such as complement component 1q (C1q) (30) are functional LAIR-1 ligands. However, it is not clear whether collagen fragments circulating in cancer patients can also ligate LAIR-1. We tested *in vitro* whether collagen fragments produced by MMPs function as LAIR-1 ligands that are able to mediate T cell suppression through LAIR-1, possibly resulting in poor prognosis of patients.

## Materials and Methods

### Bioinformatics

For survival and expression analyses, clinical data and gene expression data of TCGA samples were retrieved using UCSC Xena (31) from the UCSC Toil recompute dataset (32). Primary site samples were selected. Log2 transformed expression of selected genes (*COL1A1, MMP1, MMP9, LAIR1*) was divided into quartiles and cancer patients (**Supplementary Table 1**) were stratified based on high or low expression, corresponding to top or bottom quartile expression, respectively. When stratifying patients based on MMP, COL1A1 and LAIR1 genes, patients with the top 25% expression of all genes were selected as MMP^high^COL1A1^high^LAIR1^high^. Kaplan-Meier survival curves were estimated with the *survival* (v3.1-12) package and plotted with the *survminer* (v0.4.9) package in R (v4.0.2). Statistics were calculated by log-rank test with significance at p < 0.05. Expression data visualizations were performed using the *ggplot2* (v3.3.3) package. Selected patients based on three genes are plotted in scatter plots showing the expression of two out of three genes (**Supplementary Figure 2**). To perform correlation analysis of *PTPRC* (CD45) and *LAIR1*, log2 transformed expression of the two genes was plotted and Pearson’s correlation was determined using Base R (**Supplementary Figure 1**).

### Generation of Collagen I Fragments

Human collagen I (Sigma-Aldrich #C7774, lot #SLCD8080 and lot #SLBV1411) was reconstituted in 50 mM acetic acid (Merck #1000631000) and collagen I was dialyzed in a dialysis cassette (ThermoFisher #66333) for three days in MMP buffer, consisting of 50 mM Tris-HCl (Sigma-Aldrich #T2194-1L), 150 mM NaCl (Sigma-Aldrich #31434), 10 mM CaCl_2_ (Merck #23820500), 10 μM ZnCl_2_ (Merck #8816), 0.05% Brij-35 (Sigma-Aldrich #B4184-100ML) pH 7.5, in order to remove the acetic acid. The MMP buffer volume was 200 times the dialysis sample volume and was refreshed three times during dialysis.

p-AminoPhenylMercuric Acetate (APMA; Sigma-Aldrich #164610-700MG) was freshly reconstituted in dimethyl sulfoxide (DMSO; Sigma-Aldrich #D5879-1L-M) at 50 mM and diluted in MMP buffer to the appropriate concentration. Human MMP1 (Abcam #ab124850 for **Figures 2** and **5**, and R&D #901-MP for **Figures 3** and **4**) was activated using 1 mM APMA and human MMP9 (Abcam #ab157344) was activated using 2 mM APMA, both for 4 hours at 37°C. MMP1 was added to dialyzed collagen I in a 1:186 MMP1:collagen weight ratio, and MMP9 was added in a 1:541 MMP9:collagen weight ratio. As a control, DMSO was added to dialyzed collagen I to a final concentration of 0.16%, which is identical to the final concentration of DMSO in the MMP treated collagen I samples. All samples were prepared in NoStick low-adhesion Microcentrifuge tubes (Alpha Laboratories #LW2410) to prevent collagen I adhesion to the tubes. The samples were diluted to a final collagen I concentration of 0.744 mg/ml using MMP buffer and incubated for 24 hours at 37°C to enable fragmentation by the MMPs. To stop the MMP activity, EDTA (Calbiochem #324503) was added to a final concentration of 1 μM.

### SDS-PAGE

Samples were prepared with 4X Laemmli sample buffer (BioRad Laboratories #161-0747) supplemented with 10% β-mercaptoethanol (Sigma-Aldrich #63689) and heated for 10 minutes (min) at 85°C before loading 15 μl/well on 12-well 4-20% TGX precast protein gels (Biorad Laboratories #4561095). As a marker 2 μl of the Precision Plus Protein dual color marker (BioRad Laboratories #1610374) was loaded, and the gel was run at 45V for 3 hours.

### Silver Staining

Gels were stained using a silver staining as follows: gels were fixed using 50% methanol (Honeywell #32213) and 10% acetic acid in distilled water (dH_2_O) for 1 hour at room temperature (RT) on a shaker, and subsequently incubated in 5% methanol in dH_2_O for 30 min at RT on a shaker. Gels were washed three times for 5 min with dH_2_O, whereafter the gel was sensitized using 0.02% sodium thiosulfate (Honeywell #13479) for 2 min by shaking manually. Gels were washed three times for 1 min with dH_2_O and soaked with 11.8 mM silver nitrate (Honeywell #72031630.0025) for 30 min at RT on a shaker. Gels were washed three times for 1 min with dH_2_O and developed by manually shaking in developing solution consisting of 3% sodium carbonate (Merck #106392), 0.0005% formaldehyde (Sigma-Aldrich #252549) and 0.0004% sodium thiosulfate. Development was stopped using 1.4% EDTA disodium salt (Calbiochem #324503), and gels were imaged using a GelDoc EZ imaging system (BioRad).

### Western Blot

0.45 μm Immobilon-P PVDF membranes (Merck #IPVH00010) were activated using 100% methanol for 2 min, rinsed in blot buffer consisting of 20% ethanol and 1X Tris/Glycine buffer (BioRad #1610771), and proteins were transferred from the gel to the membrane using a mini trans-blot cell at 100 V for 1 hour. Membranes were blocked with 5% bovine serum albumin (BSA; Roche #10735094001) in TBS-T overnight at 4°C on a roller bench, and subsequently stained with biotinylated anti-MMP1 (R&D #BAF901; dilution 1:1000) or anti-MMP9 (R&D #BAF911; dilution 1:1000) primary antibodies overnight at 4°C on a roller bench. Membranes were washed three times for 5 min with TBS-T before incubation with the secondary antibody Streptavidin-HRP (Biolegend #405210; dilution 1:5000) for 1 hour at RT on a roller bench. Subsequently, membranes were washed three times for 5 min with TBS-T and once 5 min with TBS. Amersham ECL (Cytiva #RPN2236) was used for detection and imaged using a ChemiDoc gel imaging system (BioRad).

### Purification of Full-Length Alpha Chains and Fragments

Sep-Pak Plus C8 cartridges (Waters Corp #WAT036775) were rinsed with 0.1% formic acid (ThermoFisher #15650880) and 2% acetonitrile (Sigma-Aldrich #1000291000) in ddH_2_O. The mock treated or fragmented collagen I in MMP buffer was loaded on the cartridges, and subsequently fractions were eluted with ddH_2_O with 0.1% formic acid and with 0% acetonitrile (fraction 1), 25% acetonitrile (fraction 2), 50% acetonitrile (fraction 3), 75% acetonitrile (fraction 4) or 100% acetonitrile (fraction 5). Fractions were dialyzed in Slide-A-Lyzer™ MINI Dialysis Devices (ThermoFisher #11562571) into dialysis buffer, consisting of 50 mM Tris-HCl (Sigma-Aldrich #T2194-1L) and 150 mM NaCl (Sigma-Aldrich #31434) pH 7.4. Lastly, the dialyzed fractions were concentrated using a CentriVap Concentrator (VWR).

### Cell Culture

2B4 T cell hybridoma cells transduced with a NFAT-GFP reporter and human LAIR-1-CD3ζ – the human LAIR-1 reporter cells – or transduced with a NFAT-GFP reporter and CD3ζ – the wild-type (WT) reporter cells – were cultured at 37°C with 5% CO_2_ in RPMI 1640 (Life Technologies #52400025) supplemented with 10% Fetal Bovine Serum (FBS; Sigma-Aldrich #F7524, lot #BCBW0228) and 1% Penicillin/Streptomycin (Gibco #15070063; equivalent to 50 U/ml and 50 μg/ml respectively). Human LAIR-1 transfected WT reporter cells were cultured identically.

### Reporter Cell Assay

Reporter cell assays were performed as previously described (28). Briefly, black Falcon clear flat bottom 96-well plates (Corning #353219) were coated with indicated concentrations of human collagen I in MMP buffer, anti-mouse-CD3 antibody (BD #553057, lot #8068517), anti-human-LAIR-1 antibody (8A8; produced in house) or isotype control (eBiosciences #16-4714-85, lot #4347633) in Phosphate Buffered Saline (PBS; Sigma-Aldrich #D8537) by centrifuging for 3 min at 1700 rpm (514 rcf) and incubating overnight at 4°C. The next day, plates were washed with PBS, and if applicable, pre-incubated with indicated concentrations of LAIR-2-Fc (Nextcure lot #0027-NP045-045-1), LAIR-2-Fc dead (Nextcure lot #0046-NP267-094), isotype control Fc (Nextcure lot #0027-NP097-102-1) or isotype control Fc dead (Nextcure lot #0027-NP099-086-1) in culture medium by centrifuging for 5 min at 1500 rpm (250 rcf) at RT and incubating for 2 hours at 37°C.

Reporter cells were harvested and, if applicable, pre-treated with 50 μg/ml control mouse F(ab’)_2_ (SouthernBiotech #0115-14) or anti-human-LAIR-1 F(ab’)_2_ (produced in-house) for 30 min at 37°C. Cells were seeded at 1 x 10^6^ cells/ml in 50 μl/well into the medium with fusion proteins and centrifuged for 3 min at 1700 rpm (570 rcf) at RT. For IncuCyte imaging, plates were placed in the IncuCyte S3 (Sartorius) and green fluorescence of the GFP expressed by the reporter cells was imaged every hour for 24 hours at 10X magnification. For flow cytometry, plates were incubated overnight at 37°C with 5% CO_2_. The next day, cells were resuspended in FACS buffer consisting of PBS supplemented with 1% BSA, 0.01% sodium azide (NaN_3_) and 0.1 mM EDTA, and were measured on a LSRFortessa (BD Biosciences) or FACSCanto II (BD Biosciences). WT reporter cells were assessed as controls and never responded to stimulation with collagen (fragments).

### Inhibition of CD3 Signalling

Similar to the reporter cell assay, black Falcon clear flat bottom 96-well plates (Corning #353219) were co-coated with 2.5 μg/ml anti-mouse-CD3 antibody (BD #553057, lot #8068517) and indicated concentrations of collagen I by centrifuging for 3 min at 1700 rpm (514 rcf) and incubation for 3 hours at 37°C. Subsequently, plates were washed with PBS. Parental (WT) and human LAIR-1 transfected reporter cells were harvested by resuspending and seeded at 0.25 x 10^6^ cells/ml in 200 μl/well into the wells and centrifuged for 3 min at 1700 rpm (570 rcf) at RT. Plates were incubated overnight at 37°C with 5% CO_2_. The next day, cell-free supernatant was harvested, and stored at -20°C until cytokine measurements by Enzyme-Linked ImmunoSorbent Assay (ELISA). Cells were resuspended in FACS buffer consisting of PBS supplemented with 1% BSA, 0.01% sodium azide (NaN_3_) and 0.1 mM EDTA, and were measured on a FACSCanto II (BD Biosciences). Due to baseline differences in NFAT-GFP expression, inhibition of each cell line was calculated as follows:


$$\frac{\left(\text{\%GFP+ cells in CD}3+\text{BSA well})-(\text{\%GFP+ cells in CD}3+\text{collagen}(\text{fragments})\text{well}\right)}{\left(\text{\%GFP+ cells in CD}3+\text{BSA well}\right)}\times 100\%$$

### Mouse IFN-γ ELISA

Secreted mouse interferon-γ (IFN-γ) in the cell-free supernatant was measured using a mouse IFN-γ uncoated ELISA kit (Biolegend #430801) and MaxiSorp 96-wells plates (ThermoFisher #442404) following the manufacturer’s protocol. Optical density (OD) values at 450 nm and 570 nm were measured using the CLARIOstar plate reader (BMG Labtech). Due to baseline differences in IFN-γ secretion, inhibition of each cell line was calculated as follows:


$$\frac{\left(\text{IFN−}\gamma \text{ secretion in CD}3+\text{BSA well})-(\text{IFN−}\gamma \text{ secretion in CD}3+\text{collagen}(\text{fragments})\text{well}\right)}{\left(\text{IFN−}\gamma \text{ secretion in CD}3+\text{BSA well}\right)}\times 100\%$$

OD-values of 570 nm were subtracted from the 450 nm values, and these corrected values were used for ELISA analysis in SoftMax (Molecular Devices) using a 4-parameter curve fit for the standard curve. Determined IFN-γ values by SoftMax lower than the detection limit, 4 pg/ml, were manually set to 4 pg/ml to enable calculation of the inhibition and statistical analyses.

### Immune Binding Assays

Black Falcon clear flat bottom 96-well plates were coated similarly as described for the reporter cell assays, with indicated concentrations of collagen I in MMP buffer. The next day, plates were washed with PBS, and if applicable, pre-incubated with indicated concentrations of LAIR-2-Fc, LAIR-2-Fc dead, isotype control Fc or isotype control Fc dead in culture medium by centrifuging for 5 min at 1500 rpm (250 rcf) at RT and incubating for 2 hours at 37°C. Plates were washed three times with PBS and fixed with 100% ice-cold methanol for 30 min at -20°C. After washing again three times with PBS, plates were blocked for 1 hour at RT on a shaker. For the ELISA based immune binding assay plates were blocked with 1X ELISA diluent (Invitrogen #88-7066-88), and for the fluorescence based immune binding assay plates were blocked with PBS supplemented with 2% FBS and 1% normal donkey serum (Jackson ImmunoResearch #017-000-121). Plates were stained using a biotinylated LAIR-1 fusion protein (LAIR-1-Fc; produced in-house), biotinylated LAIR-2-Fc and biotinylated LAIR-2-Fc dead (all 5 μg/ml) for 1 hour at RT on a shaker. All plates were washed three times with PBS + 0.05% Tween-20 (Sigma-Aldrich #P1379). Subsequently, plates were stained with a secondary antibody for 1 hour at RT on a shaker in the dark. For the ELISA based immune binding assay a Streptavidin-HRP (Biolegend #405210; dilution 1:10,000) was used, and for the fluorescence based immune binding assay a Streptavidin-AF674 (Invitrogen #S32357; dilution 1:1000) was used. All plates were washed three times using PBS + 0.05% Tween-20. Fluorescently stained plates were imaged using the Odyssey Sa imaging system (LI-COR). ELISA based immune binding assay plates, stained with the Streptavidin-HRP, were developed using TMB substrate (Biolegend #421101) and the reaction was stopped using 1 M H_2_SO_4_ (Honeywell #07208). OD-values were measured using the CLARIOstar plate reader (BMG Labtech). The absorbance of colorimetric substrate reactions by HRP-conjugated secondary antibody binding were corrected; the OD-values of 570 nm were subtracted from the 450 nm values, and these corrected absorbance values were used.

### Data and Statistical Analyses

Raw images of silver-stained gels and of Western blots were exported. Linear image enhancement was performed in ImageJ (v1.52) using the brightness/contrast tool while visualizing over- and underexposed pixels.

Analysis of flow cytometry data was performed with FlowJo (v10.6.2), and single cells were gated based on SSC-A, FSC-A and FSC-H.

Analysis of IncuCyte images was performed using the IncuCyte 2020A analysis program (Sartorius), where green fluorescence was evaluated using Top-Hat segmentation (radius 100 μm and threshold 2 GCU), edge split turned on, minimum mean intensity of 3 GCU and an area filter of 600 μm^2^ to calculate the total green integrated intensity (GCU x µm²/Image) per well.

Immune binding assay images were quantified using the Image Studio software version 5.0 (LI-COR). Raw images were exported and used for figures without linear enhancement.

Data visualization and statistics were performed in GraphPad Prism version 8.3. The p-values lower than 0.05 were considered significant and the significant differences are indicated in each graph: * p ≤ 0.05; ** p ≤ 0.01; *** p ≤ 0.001; **** p ≤ 0.0001. Besides *, #, ‡ and ¤ are also used to indicate significance.

## Results

### Poor Overall Survival of Cancer Patients With High MMP, Collagen I and LAIR-1 Expression

**Figure 1:**
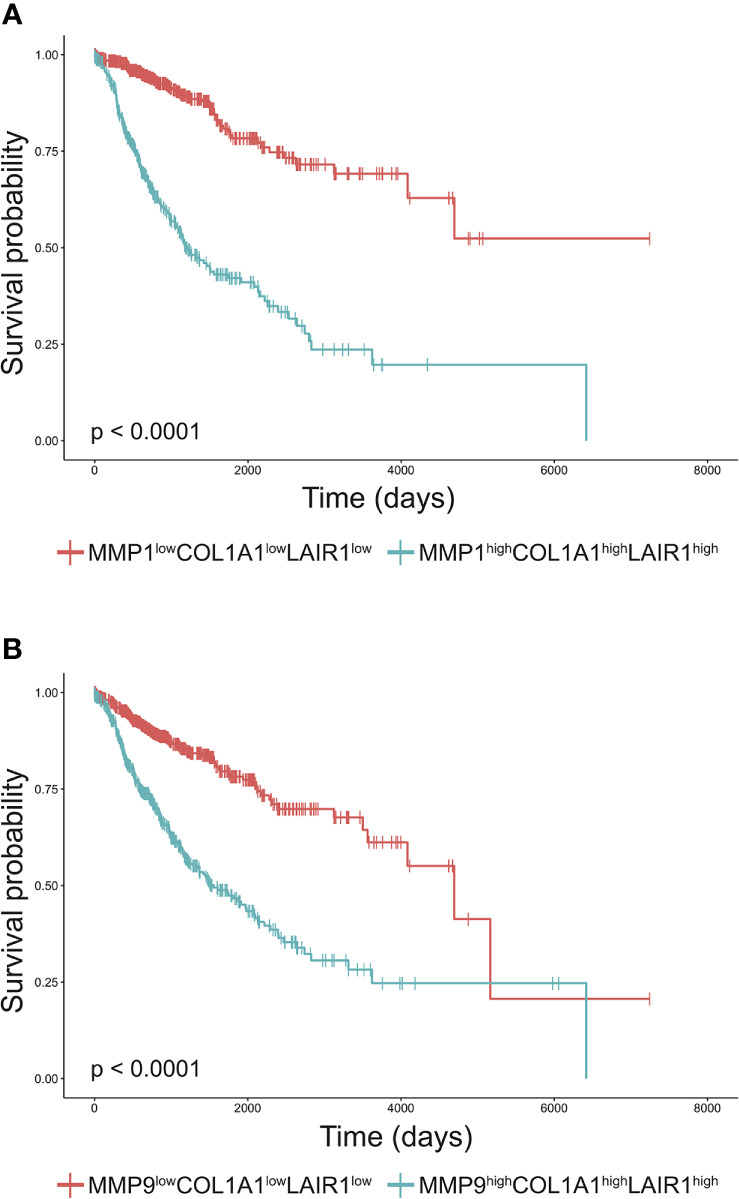
High MMP, collagen I and LAIR1 expression correlate to poor survival of cancer patients. **(A, B)** Kaplan-Meier plots of the overall-survival probability for the subgroups of patients. **(A)** Cancer patients from the TCGA database were stratified into MMP1^high^COL1A1^high^LAIR1^high^ and MMP1^low^COL1A1^low^LAIR1^low^ based on the top 25% and bottom 25% tumoral mRNA expression respectively. Similarly, patients were stratified into **(B)** MMP9^high^COL1A1^high^LAIR1^high^ and MMP9^low^COL1A1^low^LAIR1^low^. Significant differences in Kaplan-Meier plots were evaluated using a logrank test and are indicated in the bottom left corner of each graph (all p < 0.0001).

Increased tumor collagen and LAIR-1 mRNA expression are associated with worse overall survival (33). The collagen fragment C1M is generated from collagen I by MMP1 and MMP9 mediated cleavage and these MMPs are frequently overexpressed by tumors (16). To assess whether expression of these MMPs and collagen I in combination with LAIR-1 impacts survival, we performed bioinformatic analyses of tumor *MMP1, MMP9, COL1A1* and *LAIR1* mRNA expression using data from the TCGA database. LAIR-1 tumoral mRNA expression most likely originates from immune cell infiltrate in the tumor, as it correlates with CD45 tumoral mRNA expression (**Supplementary Figure 1**). When all TCGA cancer patients were stratified based on high (top 25%) or low (bottom 25%) tumoral mRNA expression of MMP1, MMP9, collagen I and LAIR-1 (**Supplementary Figure 2**), patients with MMP^high^COL1A1^high^LAIR1^high^ tumors showed significantly worse overall survival compared to patients with MMP^low^COL1A1^low^LAIR1^low^ tumors (**Figures 1A, B**). Additionally, patients with MMP^high^LAIR1^high^ tumors or MMP^high^COL1A1^high^ tumors had significantly reduced overall survival compared to patients with MMP^low^LAIR1^low^ tumors or MMP^low^COL1A1^low^ tumors respectively (**Supplementary Figure 3**). Collectively, these results suggest tumoral mRNA expression of *MMP1, MMP9, COL1A1* and *LAIR1* significantly impacts survival of cancer patients, and therefore led to the hypothesis that MMP generated collagen I fragments might activate LAIR-1.

### *In Vitro* Fragmentation of Collagen I by MMPs Generates LAIR-1 Activating Collagen Fragments

**Figure 2:**
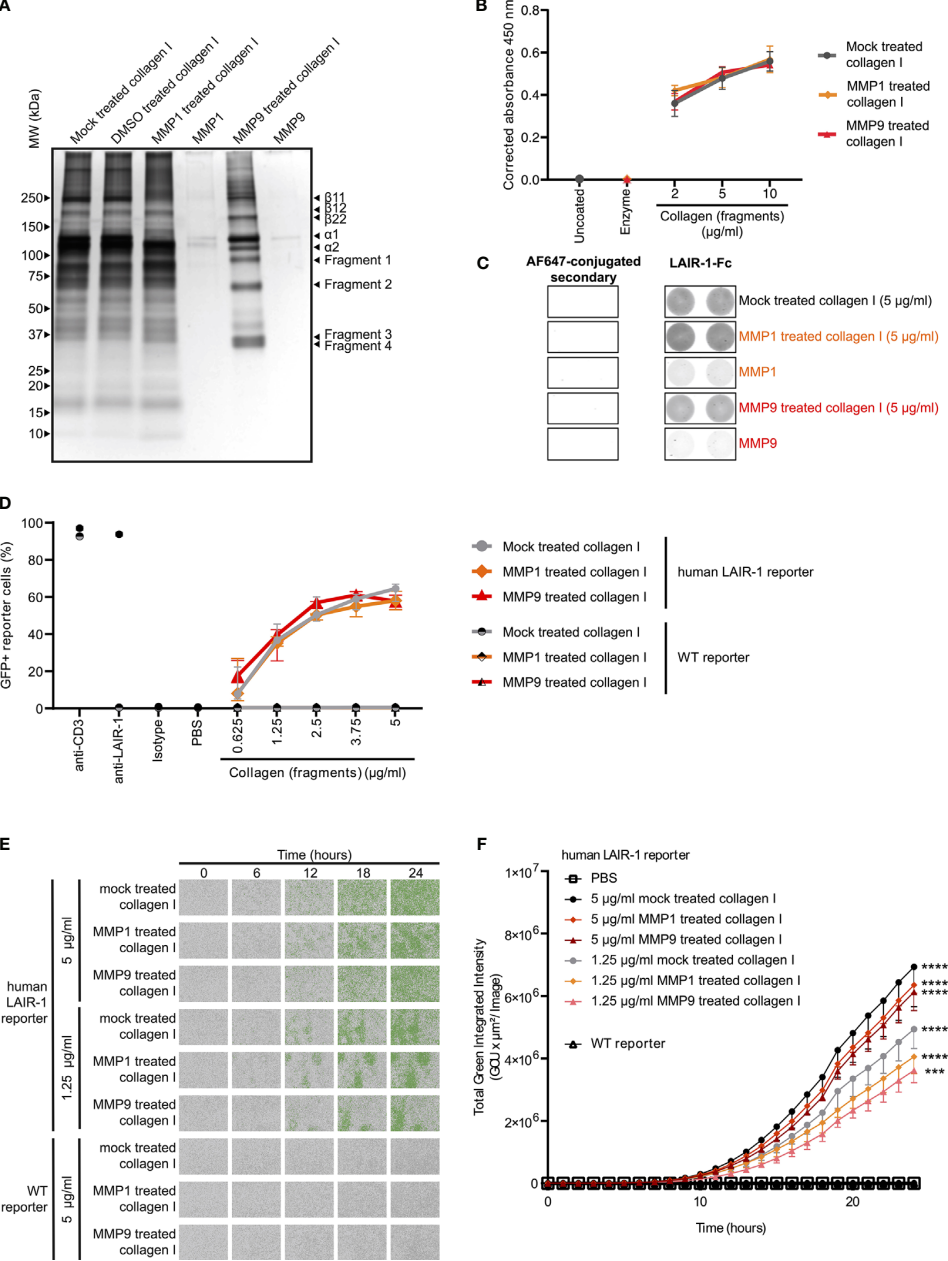
MMP1 and MMP9 generated collagen I fragments bind to and activate LAIR-1. **(A)** Analysis of MMP1 and MMP9 generated collagen I fragments by SDS-PAGE and silver staining. Representative image of n = 3. **(B, C)** LAIR-1-Fc protein binding to mock treated, MMP1 treated and MMP9 treated collagen I. **(B)** LAIR-1-Fc ELISA based immune binding assay (n = 3 with technical duplicates). **(C)** LAIR-1-Fc fluorescence based immune binding assay. Representative images show immunofluorescence by the AF647-conjugated secondary antibody alone or by secondary antibody binding to LAIR-1-Fc, representative of n = 3. **(D)** Flow cytometry analysis of NFAT-GFP reporter cells, representing LAIR-1 ligation (n = 3 with technical duplicates). Anti-human-LAIR-1 and anti-mouse-CD3 are positive controls for reporter cell activation. The isotype antibody and PBS are negative controls. **(E)** Representative images by live-cell IncuCyte imaging upon stimulation with indicated concentrations of collagen and collagen fragments (n = 3). **(F)** Quantification of the total green integrated intensity in the images from the live-cell IncuCyte imaging over time (n = 3 with technical duplicates). Significant differences at t = 24 hours compared to WT reporter cells are indicated, tested using a two-way ANOVA with Tukey’s multiple comparison correction. In all panels symbols represent the mean and whiskers indicate the standard deviation.

Type I collagen is the most abundantly expressed collagen in the human body and consists of two alpha 1 chains and one alpha 2 chain (34). Upon fragmentation of collagen I the clinically relevant collagen fragment C1M is generated, composed of two fragmented alpha 1 chains and one fragmented alpha 2 chain (19). *In vitro* cleavage of collagen I with MMP1 and MMP9 (**Supplementary Figure 4**) resulted in increased presence of fragments compared to mock treated collagen I (**Figure 2A**). Mock treated collagen I was processed identically to the MMP fragmented collagen I except for the addition of the enzymes. In particular, fragments 1 and 2 corresponded to the alpha 1 and 2 chains of C1M based on size, but additional fragments (fragments 3 and 4) were also generated by MMP1 and MMP9. LAIR-1-Fc fusion protein bound to mock treated, MMP1 treated and MMP9 treated collagen I to a similar extent, as detected by absorbance (**Figure 2B**) and by fluorescence (**Figure 2C**). We previously developed a reporter cell line expressing the extracellular domain of human LAIR-1 conjugated to CD3ζ, which induces GFP expression upon ligand interaction and receptor triggering due to the presence of an NFAT-GFP reporter (**Supplementary Figure 5**) (28). MMP1 treated and MMP9 treated collagen I dose-dependently activated human LAIR-1 reporter cells to a similar extent as mock-treated collagen I, as analyzed by flow cytometry (**Figure 2D**) and by live-cell IncuCyte imaging (**Figures 2E, F** and **Supplementary Figure 6**). Collectively, these results indicate that collagen I fragmentation by MMP1 and MMP9 does not preclude binding to and activation of LAIR-1.

### Purified Collagen I Fragments Are Functional LAIR-1 Ligands

**Figure 3:**
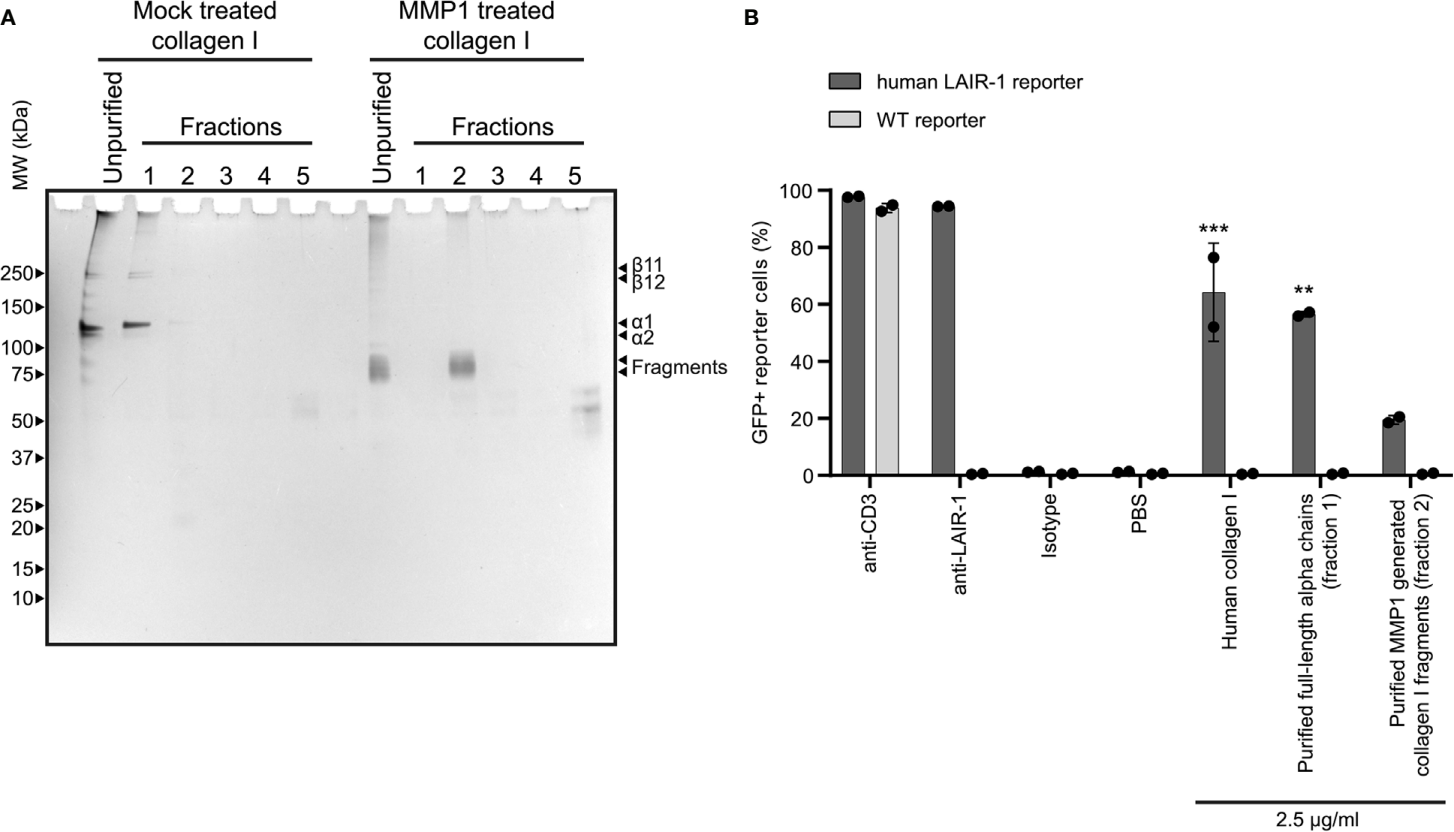
Purified collagen I fragments are functional LAIR-1 ligands. **(A)** Analysis of purified full-length alpha chains and purified MMP1 generated collagen I fragments by SDS-PAGE and silver staining. **(B)** Flow cytometry analysis of NFAT-GFP reporter cells, representing LAIR-1 ligation (n = 2). Anti-human-LAIR-1, anti-mouse-CD3 and 2.5 μg/ml human collagen I are positive controls for reporter cell activation. The isotype antibody and PBS are negative controls. Statistically significant differences compared to WT reporter cells are indicated with * (tested using two-way ANOVA with Tukey’s multiple comparison correction).

After MMP treatment, some residual alpha chains of collagen I remained unfragmented. To ensure that the activation of LAIR-1 by fragmented collagen was not solely dependent on these remaining alpha chains, we purified the fragments generated using reverse phase chromatography (**Figure 3A**) and assessed LAIR-1 ligation by purified full-length alpha chains and purified fragments. Purified MMP1 generated collagen I fragments, originating from fraction 2 of the MMP1 treated collagen I, dose-dependently activated human LAIR-1 reporter cells, although to lesser extent compared to purified full-length alpha chains originating from fraction 1 of the mock treated collagen I (**Figure 3B**). Anti-LAIR-1 F(ab’)_2_, but not control F(ab’)_2_, blocked the purified fragment-induced LAIR-1 reporter cell activation (**Supplementary Figure 7**), confirming that the reporter cell activation by purified collagen I fragments was specific for LAIR-1.

### Collagen I Fragments Inhibit T Cell Effector Function

**Figure 4:**
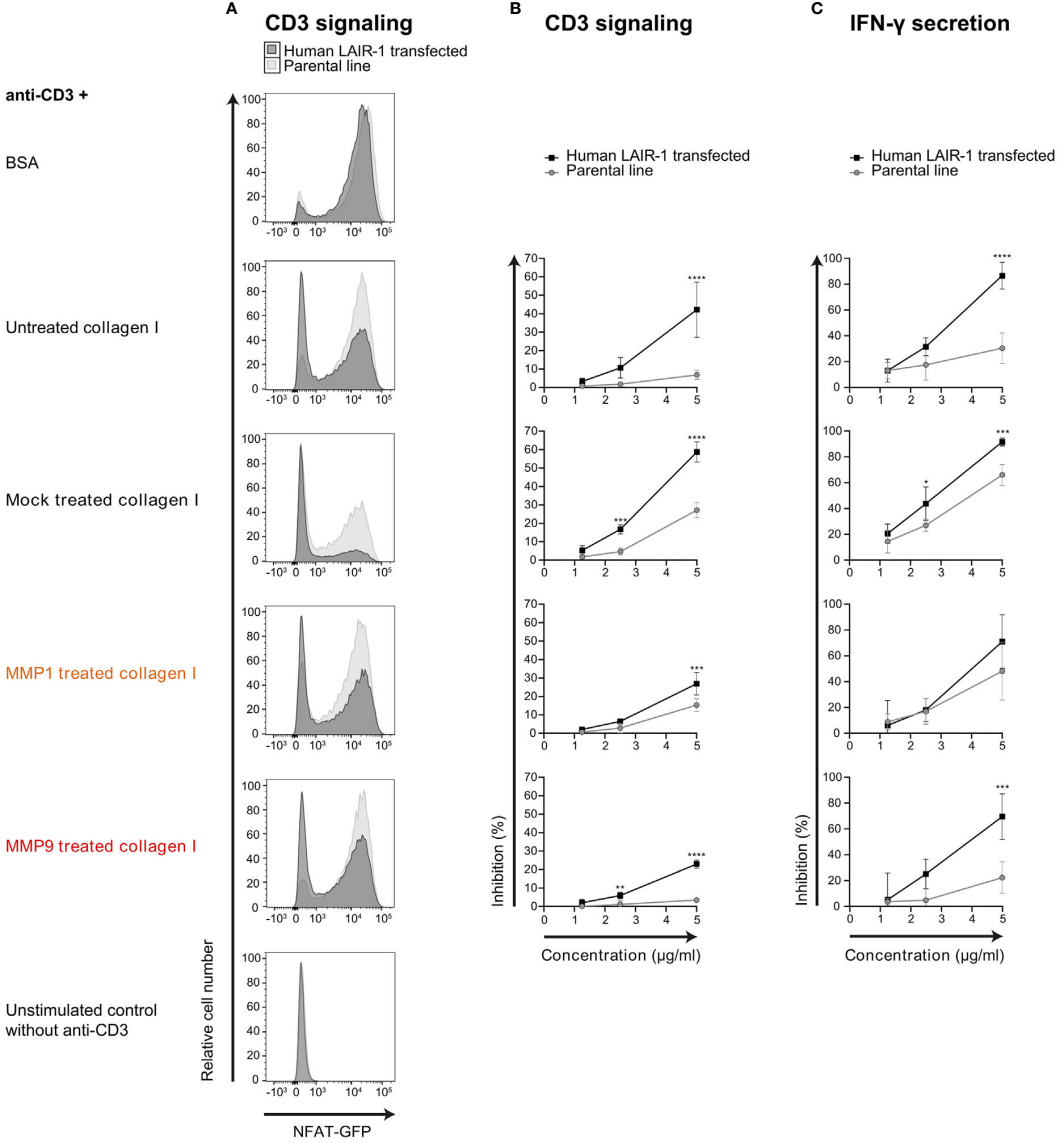
MMP1 and MMP9 generated collagen I fragments inhibit T cell function through LAIR-1. **(A)** Flow cytometry analysis of parental (light grey) or full-length human LAIR-1 expressing (dark grey) NFAT-GFP CD3 reporter cells unstimulated, and stimulated with anti-mouse-CD3 in the presence of 5 μg/ml BSA, untreated collagen I, mock treated collagen I, MMP1 treated collagen I and MMP9 treated collagen I. **(B)** Inhibition of CD3-signaling-induced GFP expression by increasing concentrations of collagen or collagen fragments compared to BSA. **(C)** Inhibition of anti-CD3 induced IFN-γ secretion by increasing concentrations of collagen or collagen fragments compared to BSA. Significant differences compared to the parental cell line are indicated, tested using a two-way ANOVA with Sidak’s multiple comparison correction.

Mouse CD3 ligation on 2B4-NFAT-GFP reporter cells results in NFAT translocation and GFP expression (35). We previously showed that ligation of both CD3 and LAIR-1 on full-length human LAIR-1 transfected NFAT-GFP reporter cells results in inhibition of CD3 signaling (36). We used this system to test the capacity of MMP fragmented collagen to inhibit TCR signaling, visualized by reduced GFP expression. Similar to full-length collagen I, both MMP1 and MMP9 derived collagen I fragments inhibited CD3 signaling (**Figures 4A, B**) and IFN-γ secretion (**Figure 4C**) of human LAIR-1 expressing reporter cells. To control for interference with anti-CD3 coating by collagen fragments, the parental NFAT-GFP reporter cells were also assessed, where we indeed observed a decreased CD3 response in the presence of high concentrations of collagen fragments. However, there still was a significant LAIR-1-mediated inhibition of CD3 signaling and, for MMP9 derived fragments, also IFN-γ secretion compared to the parental reporter cells. Altogether, this indicates that collagen I fragments inhibit T cell receptor signaling and IFN-γ secretion through LAIR-1.

### LAIR-1 Binding and Signaling by Collagen I Fragments Is Blocked by LAIR-2 Fusion Proteins

**Figure 5:**
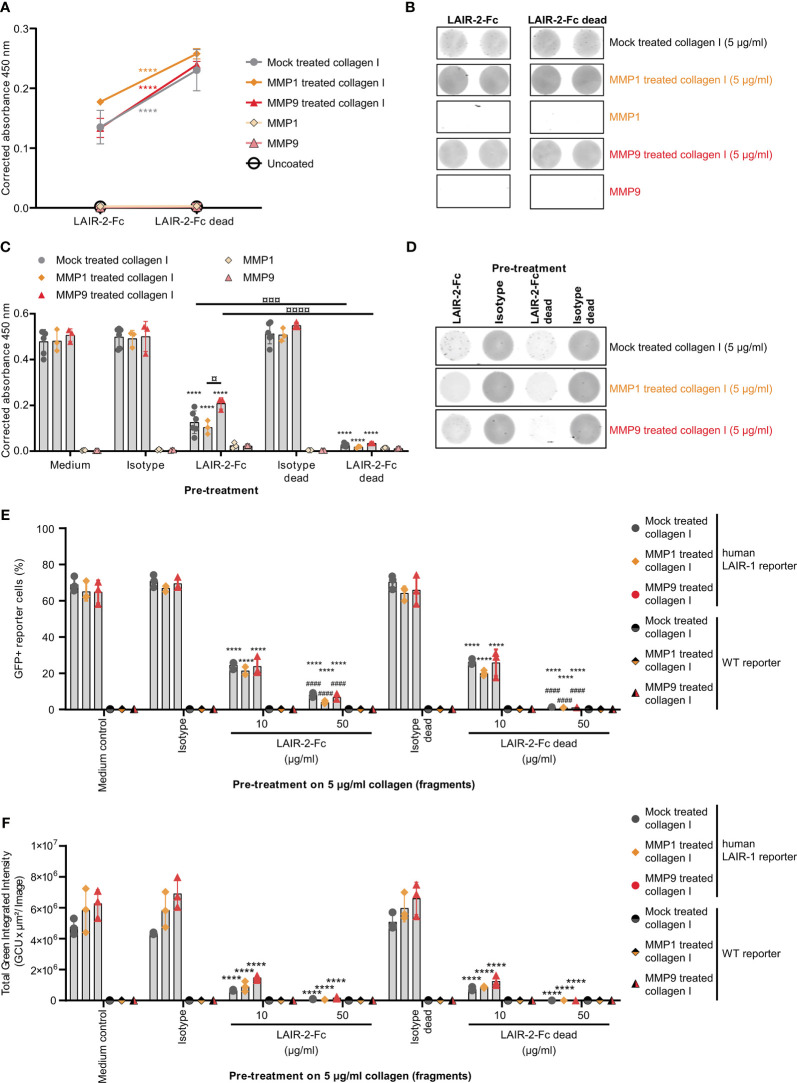
LAIR-2 fusion proteins block LAIR-1 activation by collagen I fragments. **(A)** LAIR-2-Fc and LAIR-2-Fc dead binding to 5 μg/ml coated mock treated, MMP1 treated and MMP9 treated collagen I by ELISA based immune binding assay (n = 3 with technical duplicates). Symbols represent the mean and whiskers indicate the standard deviation. **(B)** LAIR-2-Fc and LAIR-2-Fc dead binding by fluorescence based immune binding assay (representative of n = 3). **(C)** LAIR-1-Fc ELISA based immune binding assay after pre-treatment with LAIR-2-Fc and LAIR-2-Fc dead of 5 μg/ml mock treated, MMP1 treated and MMP9 treated collagen I (n = 3 with technical duplicates). **(D)** LAIR-1-Fc fluorescence based immune binding assay after pre-treatment with LAIR-2-Fc and LAIR-2-Fc dead (representative of n = 3). **(E)** Flow cytometry analysis of GFP+ reporter cells, representing LAIR-1 activation, after pre-treatment with LAIR-2 fusion proteins (n = 3 with technical duplicates). **(F)** Quantification of the total green integrated intensity in the images from the live-cell IncuCyte imaging at 24 hours upon stimulation with pre-treated 5 μg/ml coated, differently treated collagen I (n = 3 with technical duplicates). In **(C, E, F)** * indicates statistically significant differences compared to the respective isotype control (isotype for LAIR-2-Fc and isotype dead for LAIR-2-Fc dead). # indicates statistically significant differences compared to the respective 10 μg/ml pre-treatment. In all panels, significance is tested using a two-way ANOVA with Tukey’s multiple comparison correction.

To prevent immunosuppression through LAIR-1, the interaction with its ligand – collagen – must be prevented by blocking either the receptor or the ligand. As previously described, dimeric LAIR-2-Fc proteins are able to block LAIR-1-collagen interactions, resulting in decreased tumor outgrowth in humanized mouse models (33, 37). LAIR-2-Fc and LAIR-2-Fc dead are effective blockers of LAIR-1 activation by full-length collagen (33). LAIR-2-Fc, with a functional IgG1 Fc-tail, and LAIR-2-Fc dead, with a silenced IgG1 Fc-tail, both bound to mock treated and fragmented collagen I, with increased binding of LAIR-2-Fc dead compared to LAIR-2-Fc (**Figures 5A, B**). Pre-treatment of collagen I fragments with LAIR-2 fusion proteins decreased LAIR-1 binding, where LAIR-2-Fc dead pre-treatment decreased LAIR-1 binding to an even greater extent compared to LAIR-2-Fc (**Figures 5C, D**). Additionally, LAIR-2 fusion protein pre-treatment of collagen I fragments reduced the percentage of GFP+ LAIR-1 reporter cells dose-dependently (**Figures 5E, F** and **Supplementary Figure 8**). Collectively, these results indicate that LAIR-2-Fc and LAIR-2-Fc dead pre-treatment of MMP1 and MMP9 treated collagen I blocks LAIR-1 binding and LAIR-1 activation comparable to LAIR-2 fusion protein pre-treatment of mock treated, full-length collagen I.

## Discussion

The ECM undergoes a dramatic transformation during tumor development due to tissue remodeling (38). Changes in collagen expression, density and stiffness correlate with worse prognosis of cancer patients (3), possibly because this provides a physical barrier for immune cells, limits drug diffusion, and could induce immunosuppression (4), all underscoring the important role of collagen in cancer progression. T cells play a key role in immunity against cancer and dysfunction of T cells by the TME hampers the anti-tumor activity (27). In this study we showed that MMP generated collagen I fragments are functional LAIR-1 ligands that impair T cell function, potentially suppressing anti-tumor responses both locally and systemically in cancer patients.

ECM signatures (39), increased collagen expression (40) and increased expression of collagen-crosslinking enzymes (41) associate with poor prognosis of cancer patients. MMP generated collagen fragments are abundant in cancer patients and correlate with poor prognosis (20, 21). Collagen fragments also seem to contribute to therapeutic resistance since high levels of C1M in the circulation associate with poor response to anti-CTLA-4 therapy (22), and increased collagen turnover is associated with poor survival after anti-PD-1 therapy (42). Furthermore, LAIR-1-collagen interaction has been implicated in mediating resistance to current anti-PD-1 immunotherapy (26).

Using publicly available TCGA data we showed that cancer patients with MMP^high^COL1A1^high^LAIR1^high^ tumoral mRNA expression have worse overall survival compared to patients with MMP^low^COL1A1^low^LAIR1^low^ tumors. In our analyses, cancer patients with high MMP1 or MMP9 tumoral mRNA expression showed worse survival, in agreement with previous studies in which protein expression of MMP1 (43) and MMP9 (44) was associated with poor survival of breast cancer patients. The association of C1M levels with poor prognosis was previously explained by increased ECM degradation in the tumor (45). We now extend these findings by showing that high expression of LAIR-1 in the TME together with high MMP and collagen I expression may significantly impact survival.

The collagen I fragments we generated by MMP1 and MMP9 mediated cleavage are similar to fragments described in literature. MMP1 cleaves collagen I at the unique site between amino acids 775 and 776 (46), resulting in collagen fragmentation into two fragments: a 3/4-length fragment and a 1/4-length fragment (46, 47). MMP9 cleavage also generates 3/4-length and 1/4-length fragments by cleaving in the same region (48), although the exact cleavage site on collagen I is uncharacterized. The MMP generated 3/4-length collagen I fragment is defined as C1M (19). Cleavage of both collagen I alpha chains would result in four fragments of alpha chains on SDS-PAGE gels, as both the 3/4-length fragment and the 1/4-length fragment consist of two alpha 1 chains and one alpha 2 chain. MMP mediated fragmentation of collagen I indeed resulted in four distinct MMP generated fragments in comparison to the mock treated and DMSO treated collagen I. However, some fragmentation occurred in the mock treated collagen I sample. Since neither MMP1 nor MMP9 was present in the mock treated collagen I sample, the fragments observed likely resulted from the thermal instability of collagen I at 37°C (49).

Collagen fragments of 30-75 kDa, similar to the fragment sizes found in this study, were previously shown to inhibit osteoclast formation in a LAIR-1-dependent manner (50), while fragments that were smaller were unable to inhibit osteoclast formation. Therefore, we hypothesize that the fragment length determines the capacity to activate LAIR-1, possibly because a larger fragment size facilitates more efficient receptor clustering and induction of LAIR-1 signaling.

We observed that the purified collagen I fragments activated LAIR-1 to a lesser extent than purified full-length alpha chains. However, the use of acetonitrile during the reverse phase chromatography to isolate the fragments could have partially denatured the fragments, as partial collagen denaturation by 25% acetonitrile has been previously described (51). The full-length alpha chains were not exposed to acetonitrile, and LAIR-1 activation was comparable to the untreated collagen control. Despite this, the collagen fragments were still able to activate LAIR-1, and thus are true activating LAIR-1 ligands, but the extent to which the fragments activate LAIR-1 compared to full-length collagen alpha chains *in vivo* remains to be determined.

Besides size, the collagen structure could also affect LAIR-1 activation. Collagen is a right-handed, tight, triple helix with hydroxyprolines to maintain the helical conformation (11). The collagen structure is important for recognition by LAIR-1, as GPO triplets that are present in collagen are important – although not the only determinant – for LAIR-1 binding to collagen (36). MMP mediated fragmentation of collagen could alter the structure of the collagen, potentially generating more or less LAIR-1 binding sites.

Collagen present in the TME modulates immune cell functions by binding to several receptors, including LAIR-1 (24, 52, 53). LAIR-1 signaling has previously been shown to inhibit T cell function (24, 52, 53) and LAIR-1 expressing T cells are important players in the anti-tumor immune response (54, 55). Since LAIR-1 also inhibits cell function of other immune cells, such as dendritic cells (30), macrophages (56) and NK cells (24), the interaction of collagen fragments with other immune cell subsets besides T cells might also impact their function, and interaction with T cells, both in the TME and in the circulation. In the future, the effect of collagen fragments on human primary T cells, and their interaction with other immune cell populations, would address the role of collagen fragments in a more relevant setting to cancer patients.

Recent clinical and preclinical studies have shown that systemic immune perturbations occur during tumor development and that peripheral immune cells have a crucial contribution to anti-tumor immune responses (57). Tumor derived factors, such as collagen fragments, can act locally within the TME, but also spread systemically *via* the circulation mediating distant effects (e.g. on bone marrow, lymph nodes and spleen) (58, 59). We now show that collagen fragments are LAIR-1 ligands, possibly suggesting that systemic immunosuppression might occur *via* circulating collagen fragments that mediate LAIR-1 activation. Nonetheless, whether systemic immunosuppression in cancer is indeed mediated by collagen fragments *via* LAIR-1 should be further investigated.

In this study a limited number of MMPs were used and only fragmented collagen I was assessed in relation to LAIR-1 mediated immunosuppression. However, several other MMPs and proteases are known, each with different substrates and different cleavage sites (60), thereby generating a range of collagen fragments, all with unique characteristics and potential (LAIR-1) binding sites. Additionally, MMP mediated cleavage of collagen could reveal cryptic domains with novel functions that are hidden in the folded, full-length molecule (61). These fragments could have novel effects on collagen-receptors, such as LAIR-1. It would also be important to study collagen fragments generated from other collagens upregulated in the TME in relation to LAIR-1 activation. Since proteolytic degradation of ECM components can be both pro- and anti-tumorigenic (62, 63), a more detailed profiling of the generated collagen fragments could lead to the development of an ECM signature with potential diagnostic and/or therapeutic application.

Here, we have shown that MMP generated collagen fragments cause immunomodulation through LAIR-1, potentially mediating T cell immunosuppression both in the TME and in the circulation. These findings further strengthen LAIR-1 as a promising therapeutic target to improve cancer treatment and overall survival (26, 33, 37). Previously, we showed that LAIR-2-Fc can revert collagen-induced LAIR-1 mediated immunosuppression, resulting in decreased tumor outgrowth in humanized mouse models (33). Therefore, LAIR-2-Fc is currently being evaluated in a clinical trial (NCT04408599). Importantly, we have now demonstrated that LAIR-2 fusion proteins counteract the effects of MMP generated collagen fragments as well, suggesting that LAIR-2 fusion protein administration might reinvigorate T cells in the circulation and/or in the TME. Stratifying patients based on MMP generated collagen fragments could possibly guide LAIR-1 blockade therapy. Overall, our study shows that collagen fragments produced in cancer can mediate LAIR-1 induced T cell suppression, and that blocking this interaction with LAIR-2 fusion proteins could be of importance for cancer treatment.

## Data Availability Statement

The raw data supporting the conclusions of this article will be made available by the authors, without undue reservation.

## Author Contributions

LM and MR conceptualized the study. SV, LM, and MR were involved in the design of the experiments. SV and AS performed bioinformatic analyses. RC, DF, SL, and LB provided essential reagents. SV, EM-E, and JM carried out the experiments. SV, AS, LM, and MR performed analysis and interpretation of data, and wrote the manuscript. All authors were involved in critically revising the manuscript. All authors contributed to the article and approved the submitted version.

## Funding

LM is supported by the Netherlands Organization for Scientific Research (NWO) (Vici 918.15.608).

## Conflict of Interest

RC, DF, and SL are employees from Nextcure. Nextcure holds a patent on NC410. (PCT/US20 17/0453 10). LB is employed by Polpharma Biologics.

The remaining authors declare that the research was conducted in the absence of any commercial or financial relationships that could be construed as a potential conflict of interest. 

## Publisher’s Note

All claims expressed in this article are solely those of the authors and do not necessarily represent those of their affiliated organizations, or those of the publisher, the editors and the reviewers. Any product that may be evaluated in this article, or claim that may be made by its manufacturer, is not guaranteed or endorsed by the publisher.
