# Supplementary material for: Collagen Fragments Produced in Cancer Mediate T Cell Suppression Through Leukocyte-Associated Immunoglobulin-Like Receptor 1
Source: Front Immunol. 2021 Oct 7;12:733561. doi: 10.3389/fimmu.2021.733561 (PMC8529287; doi:10.3389/fimmu.2021.733561)
Supplement: Supplementary file 1 [file DataSheet_1.pdf]

Supplementary Material

1 Supplementary Figures and Tables

1.1 Supplementary Figures

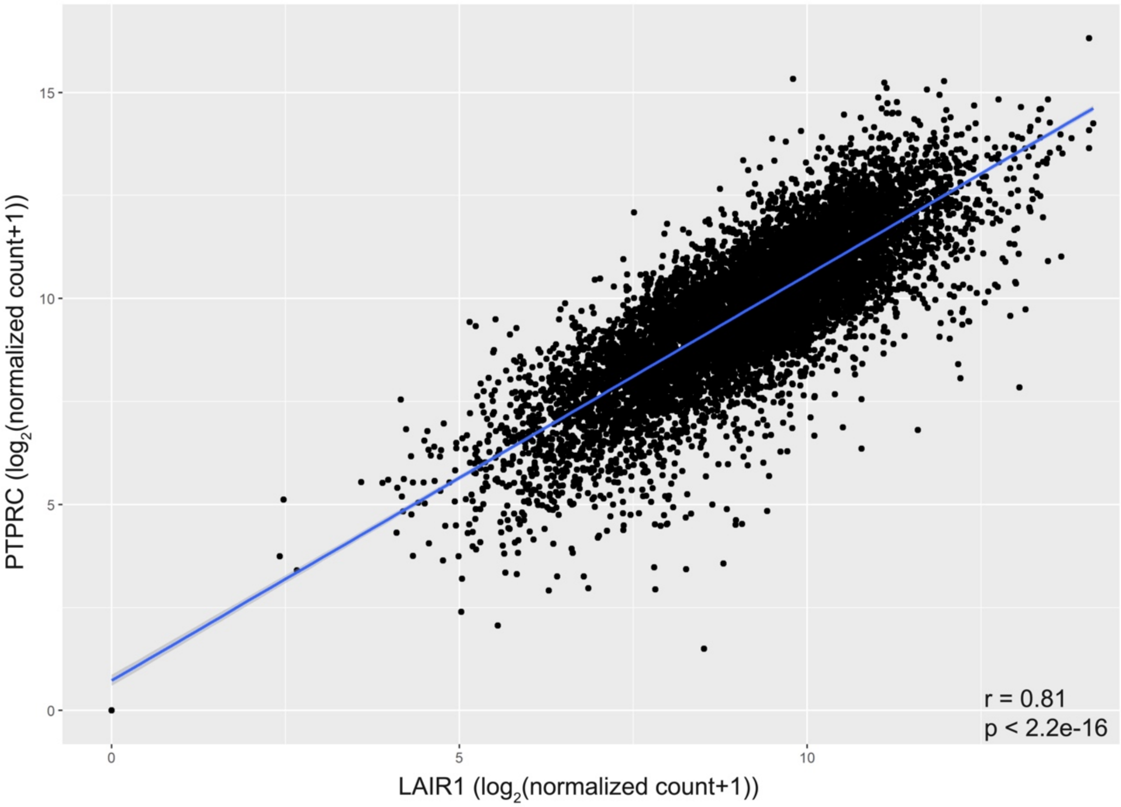

Supplementary figure 1: *PTPRC* and *LAIR1* tumoral mRNA expression correlate. Pearson's correlation between *PTPRC* (CD45) and *LAIR1* tumoral mRNA expression. Pearson's correlation coefficient and p-value are indicated in the bottom right corner of the graph.

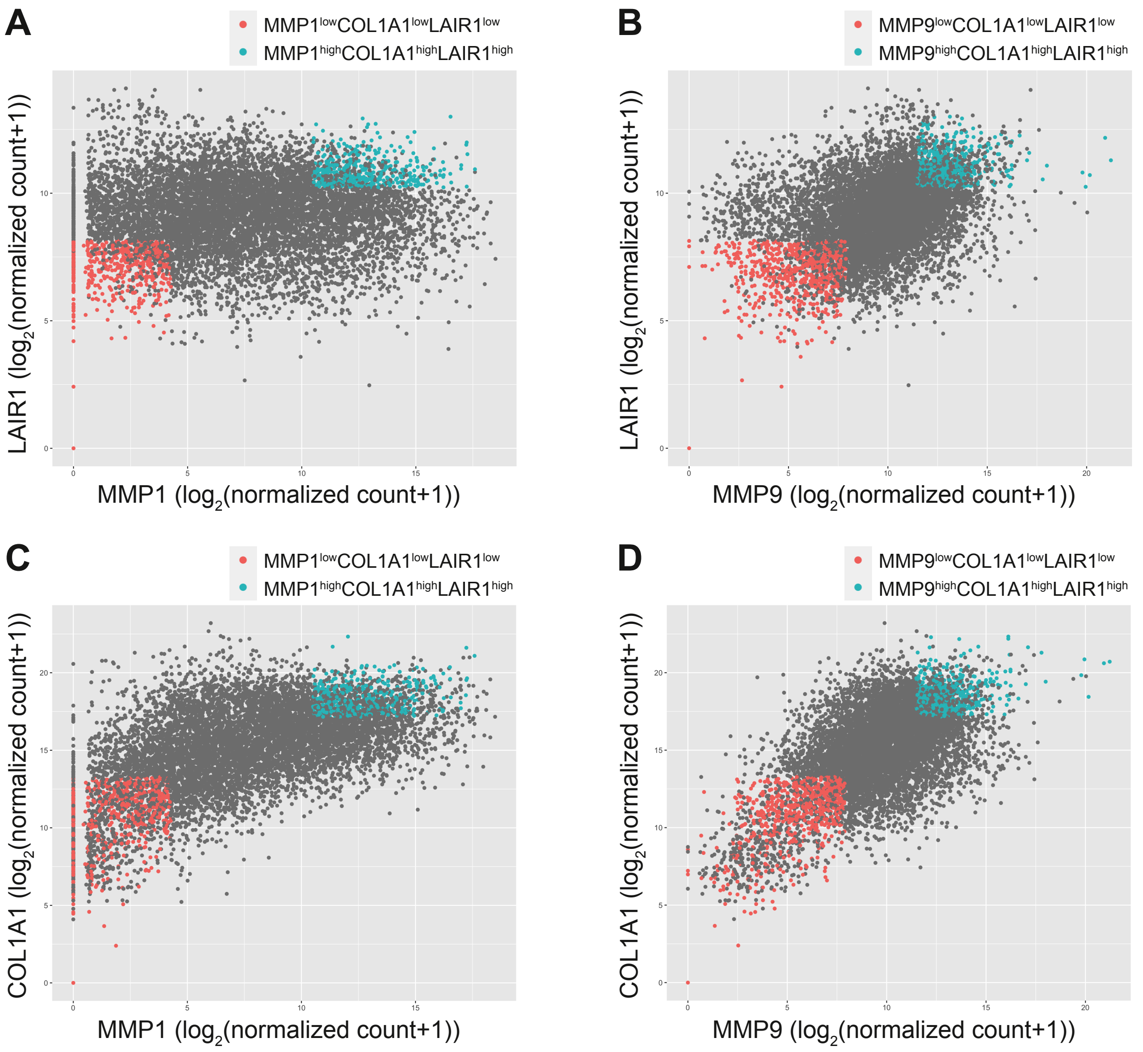

Supplementary figure 2: selection of patients from the TCGA based on *MMP1*, *MMP9*, *COL1A1* and *LAIR1* expression. A-B) Expression of **(A)** *MMP1* and *LAIR1*, **(B)** *MMP9* and *LAIR1*, **(C)** *MMP1* and *COL1A1*, and **(D)** *MMP9* and *COL1A1* showing the selected patients for figure 1.

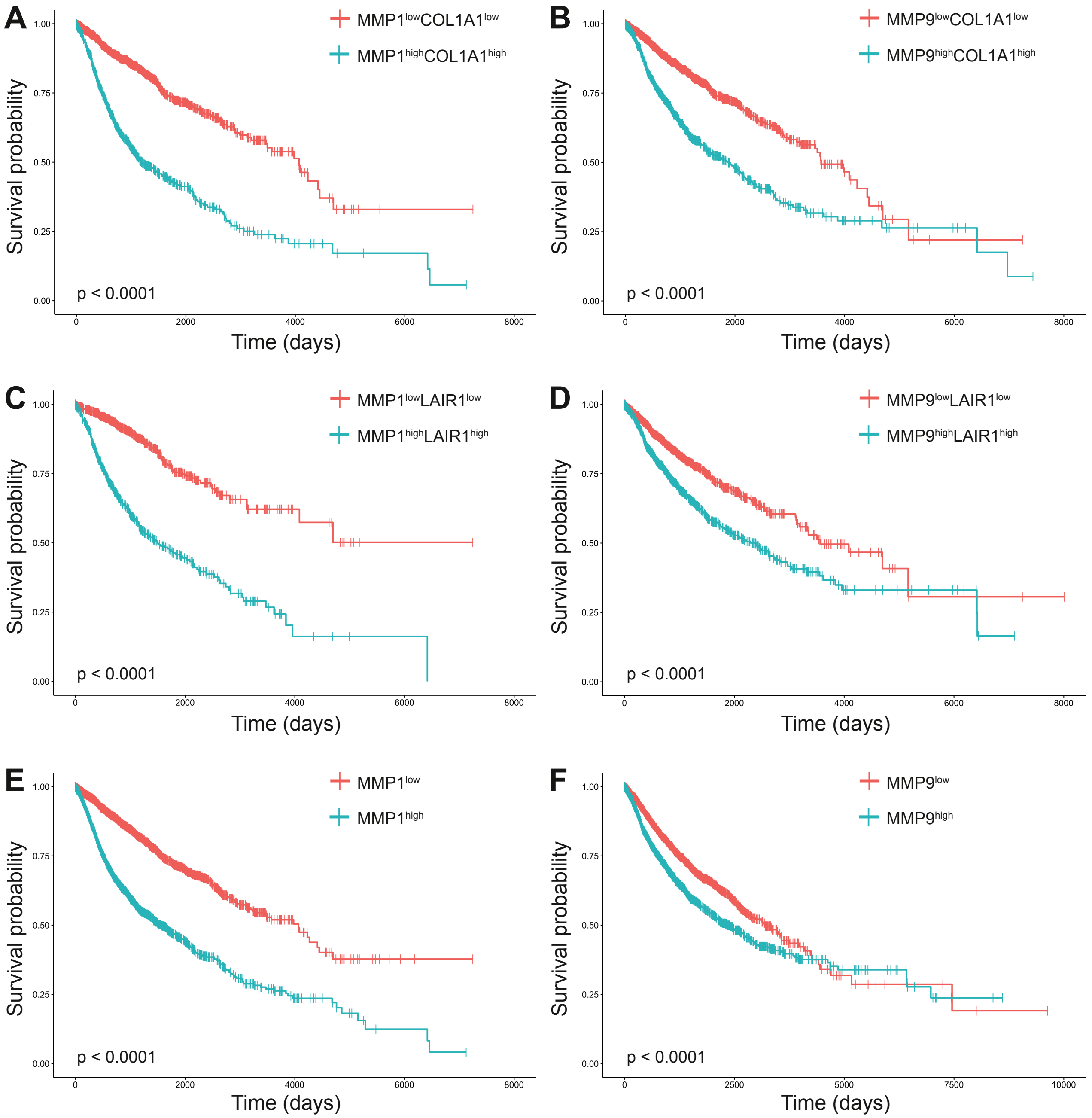

Supplementary figure 3: high MMP and LAIR1 expression, high MMP and collagen I expression, and high MMP expression correlate to poor survival of cancer patients. A-F) Kaplan-Meier plots of the overall-survival probability for the subgroups of patients. **(A)** Cancer patients from the TCGA database were stratified into MMP1^{high}COL1A1^{high} and MMP1^{low}COL1A1^{low}. Similarly, patients were stratified into **(B)** MMP9^{high}COL1A1^{high} and MMP9^{low}COL1A1^{low}, **(C)** MMP1^{high}LAIR1^{high} and MMP1^{low}LAIR1^{low} and **(D)** MMP9^{high}LAIR1^{high} and MMP9^{low}LAIR1^{low}, **(E)** MMP1^{high} and MMP1^{low}, and **(F)** MMP9^{high} and MMP9^{low} cancer patients. High and low expression of *MMP1*, *MMP9*, *COL1A1* and *LAIR1* was based on the top and bottom 25% tumoral mRNA expression respectively. Significant differences in Kaplan-Meier plots were evaluated using a logrank test and are indicated in the bottom left corner of each graph.

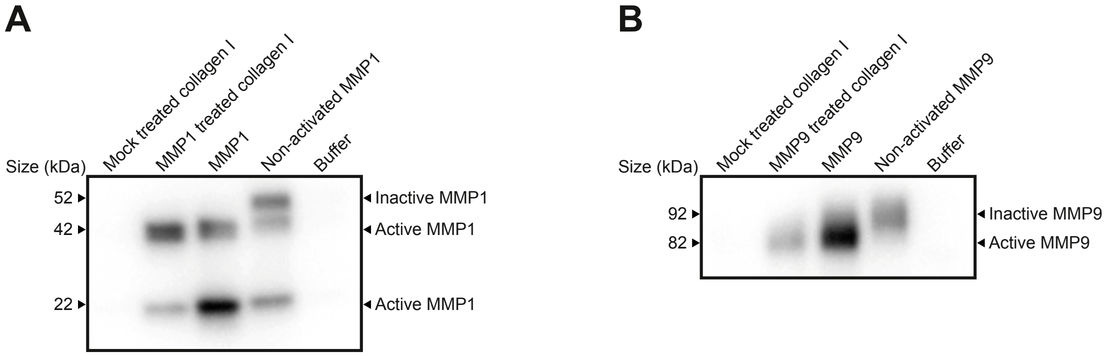

Supplementary figure 4: MMP1 and MMP9 are activated to cleave collagen I. A-B) Analysis of **(A)** MMP1 activation and **(B)** MMP9 activation by SDS-PAGE and Western blot (representative of n = 3).

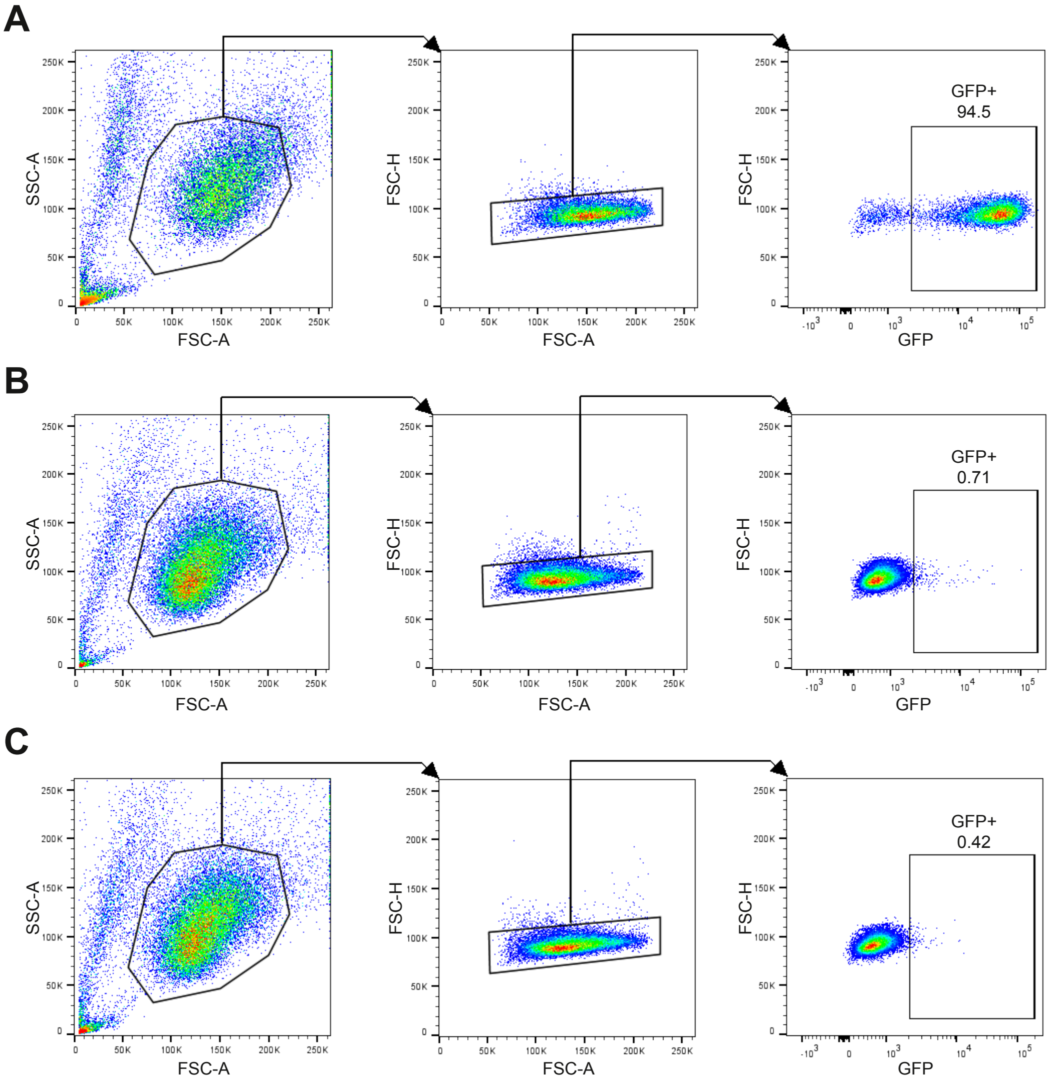

Supplementary figure 5: NFAT-GFP reporter cells. **A-C)** Gating strategies of flow cytometry experiments showing **(A)** human LAIR-1 reporter cells upon anti-human-LAIR-1 antibody stimulation, **(B)** human LAIR-1 reporter cells upon PBS stimulation and **(C)** WT reporter cells upon anti-human-LAIR 1 antibody stimulation.

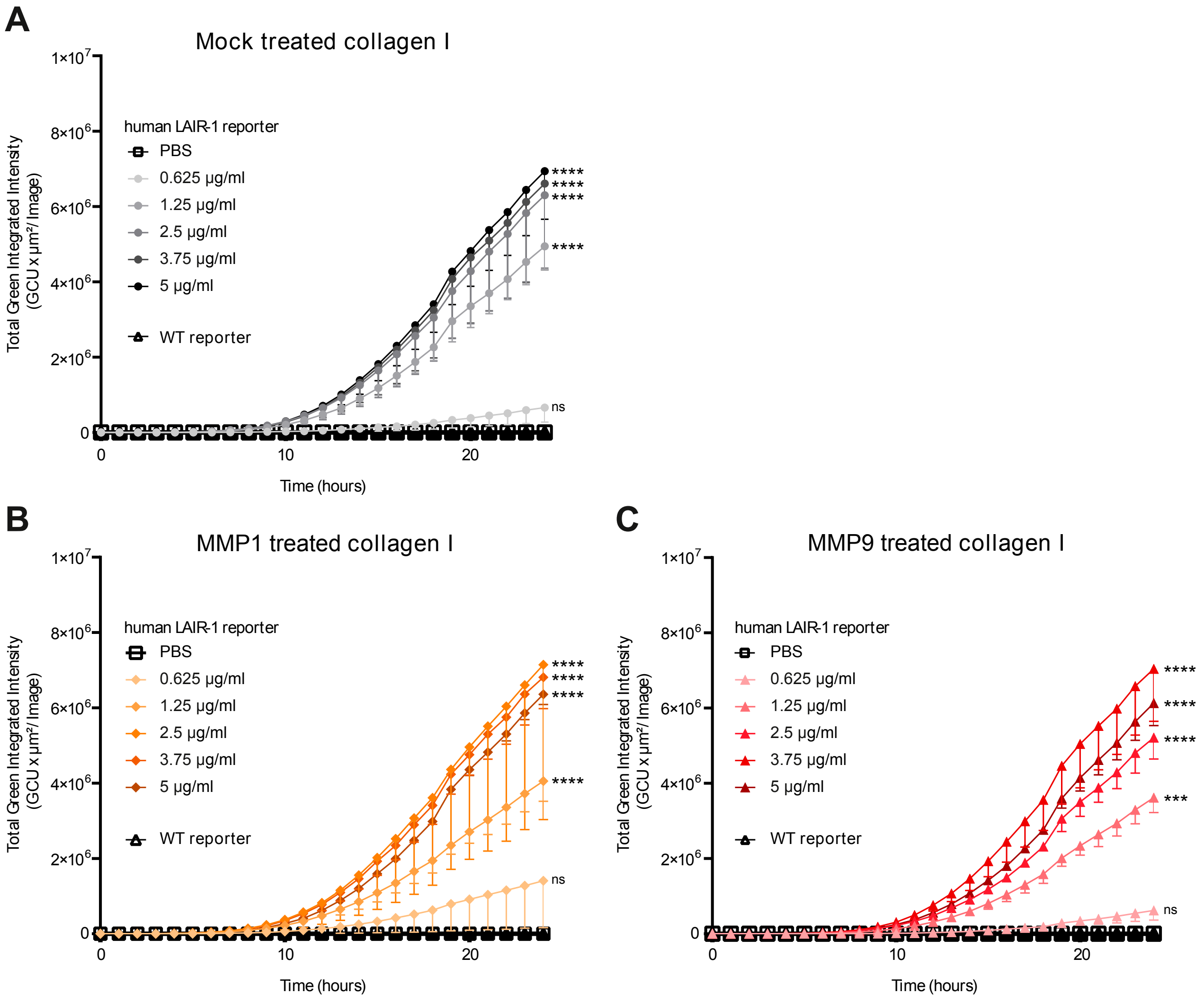

Supplementary figure 6: LAIR-1 is activated over time by mock treated, MMP1 treated and MMP9 treated collagen I. A-C) Quantification of the total green integrated intensity in the images from the live-cell IncuCyte imaging over time of increasing concentrations of **(A)** mock treated collagen I, **(B)** MMP1 treated collagen I and **(C)** MMP9 treated collagen I coated at indicated concentrations (n = 3 with technical duplicates). Statistically significant differences at t = 24 hours compared to WT reporter cells are indicated (tested using two-way ANOVA with Tukey's multiple comparison correction). In all panels symbols represent the mean and whiskers indicate the standard deviation.

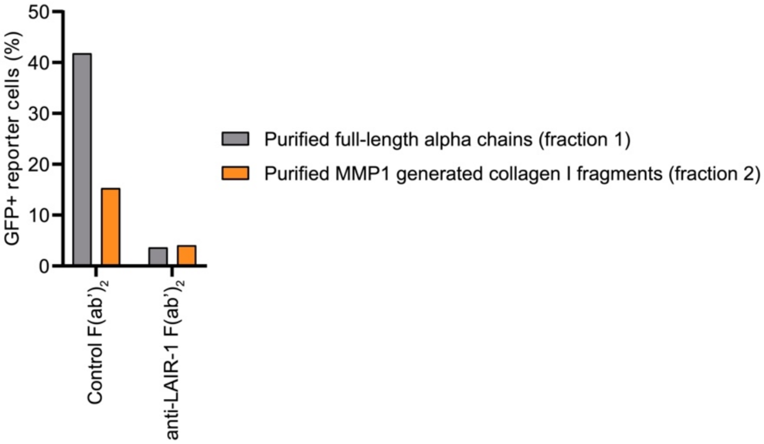

Supplementary figure 7: anti-LAIR-1 F(ab')$_2$ fragments prevent LAIR-1 activation by purified MMP1 generated collagen I fragments . Flow cytometry analysis of NFAT-GFP human LAIR-1 reporter cells pre-treated with F(ab')$_2$ fragments (n = 1) on 2.5 µg/ml purified full-length alpha chains (originating from fraction 1) and purified MMP1 generated collagen I fragments (originating from fraction 2).

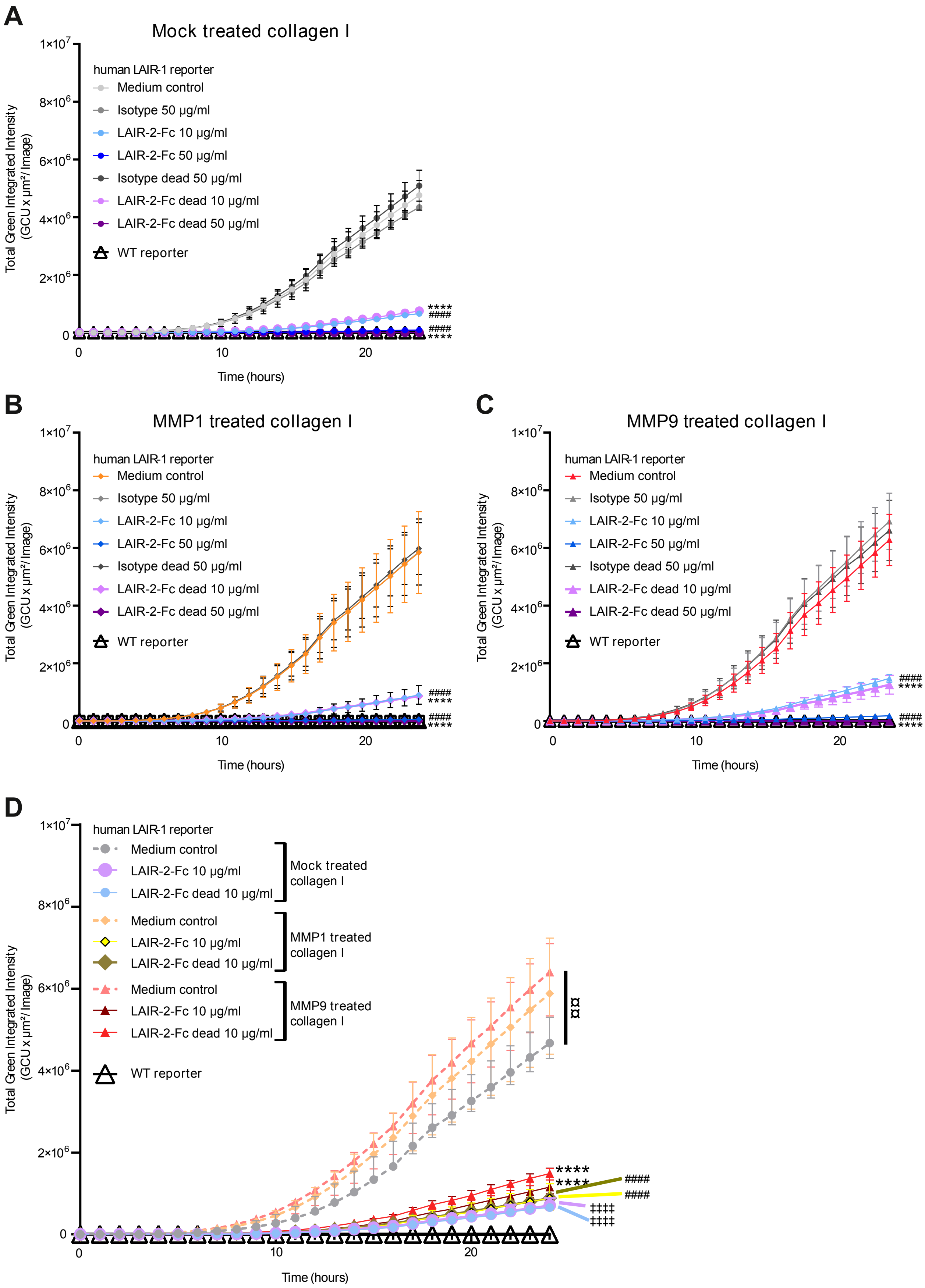
A
Mock treated collagen I
Total Green Integrated Intensity (GCU x µm²/ Image)
human LAIR-1 reporter
Medium control
Isotype 50 µg/ml
LAIR-2-Fc 10 µg/ml
LAIR-2-Fc 50 µg/ml
Isotype dead 50 µg/ml
LAIR-2-Fc dead 10 µg/ml
LAIR-2-Fc dead 50 µg/ml
WT reporter
Time (hours)
****
####
####
****
B
MMP1 treated collagen I
Total Green Integrated Intensity (GCU x µm²/ Image)
human LAIR-1 reporter
Medium control
Isotype 50 µg/ml
LAIR-2-Fc 10 µg/ml
LAIR-2-Fc 50 µg/ml
Isotype dead 50 µg/ml
LAIR-2-Fc dead 10 µg/ml
LAIR-2-Fc dead 50 µg/ml
WT reporter
Time (hours)
####
****
####
****
C
MMP9 treated collagen I
Total Green Integrated Intensity (GCU x µm²/ Image)
human LAIR-1 reporter
Medium control
Isotype 50 µg/ml
LAIR-2-Fc 10 µg/ml
LAIR-2-Fc 50 µg/ml
Isotype dead 50 µg/ml
LAIR-2-Fc dead 10 µg/ml
LAIR-2-Fc dead 50 µg/ml
WT reporter
Time (hours)
####
****
####
****
D
Total Green Integrated Intensity (GCU x µm²/ Image)
human LAIR-1 reporter
Medium control
LAIR-2-Fc 10 µg/ml
LAIR-2-Fc dead 10 µg/ml
Mock treated collagen I
Medium control
LAIR-2-Fc 10 µg/ml
LAIR-2-Fc dead 10 µg/ml
MMP1 treated collagen I
Medium control
LAIR-2-Fc 10 µg/ml
LAIR-2-Fc dead 10 µg/ml
MMP9 treated collagen I
WT reporter
Time (hours)
¤¤
****
****
####
####
‡‡‡‡
‡‡‡‡

Supplementary figure 8: LAIR-1 activation is dose-dependently prevented by LAIR-2-Fc and LAIR-2-Fc dead on mock treated, MMP1 treated and MMP9 treated collagen I. (A-C) Quantification of the total green integrated intensity in the images from the live-cell IncuCyte imaging over time of LAIR-2-Fc and LAIR-2-Fc dead pre-incubation on 5 µg/ml coated **(A)** mock treated collagen I, **(B)** MMP1 treated collagen I and **(C)** MMP9 treated collagen I. Statistically significant differences compared to the respective isotype control of the human LAIR-1 reporter at t = 24 hours are indicated. # and * indicate significant differences of LAIR-2-Fc and LAIR-2-Fc dead respectively. **(D)** Quantification of the total green integrated intensity in the images from the live-cell IncuCyte imaging at 24 hours upon stimulation with pre-treated 5 µg/ml coated, differently treated collagen I (n = 3 with technical duplicates). ¤ indicates the statistically significant difference between medium controls, and ‡, # and * indicate the difference compared to the medium control of mock treated, MMP1 treated and MMP9 treated collagen I respectively. In all panels statistics were tested using two-way ANOVA with Tukey's multiple comparison correction and in all panels symbols represent the mean and whiskers indicate the standard deviation.

1.2 Supplementary Tables

Supplementary table 1: TCGA studies used for the bioinformatic analyses of tumoral mRNA expression in relation to patient survival. The number of samples included in total, in the MMP1^{high}COL1A1^{high}LAIR1^{high} group and in the MMP9^{high}COL1A1^{high}LAIR1^{high} group is indicated per cancer type.

TCGA study abbreviation	cancer type	samples included in total	samples in MMP1^{high}COL1A1^{high}LAIR1^{high} group	samples in MMP9^{high}COL1A1^{high}LAIR1^{high} group
ACC	Adrenocortical Cancer	77	2	1
BLCA	Bladder Urothelial Carcinoma	406	23	23
BRCA	Breast Invasive Carcinoma	1090	73	118
CESC	Cervical & Endocervical Cancer	304	0	0
CHOL	Cholangiocarcinoma	36	0	0
COAD	Colon Adenocarcinoma	286	14	16
DLBC	Diffuse Large B-Cell Lymphoma	47	2	3
ESCA	Esophageal Carcinoma	181	14	12
GBM	Glioblastoma Multiforme	152	1	2
HNSC	Head & Neck Squamous Cell Carcinoma	517	33	33
KICH	Kidney Chromophobe	65	1	1
KIRC	Kidney Clear Cell Carcinoma	530	3	13
KIRP	Kidney Papillary Cell Carcinoma	287	0	0
LGG	Brain Lower Grade Glioma	507	0	0
LIHC	Liver Hepatocellular Carcinoma	368	0	2
LUAD	Lung Adenocarcinoma	504	46	56
LUSC	Lung Squamous Cell Carcinoma	492	62	55
MESO	Mesothelioma	86	3	15
OV	Ovarian Serous Cystadenocarcinoma	417	8	36

PAAD	Pancreatic Adenocarcinoma	178	43	30
PCPG	Pheochromocytoma & Paraganglioma	177	0	2
PRAD	Prostate Adenocarcinoma	495	0	1
READ	Rectum Adenocarcinoma	91	1	3
SARC	Sarcoma	258	14	42
SKCM	Skin Cutaneous Melanoma	102	2	2
STAD	Stomach Adenocarcinoma	408	69	46
TGCT	Testicular Germ Cell Tumor	132	0	10
THCA	Thyroid Carcinoma	504	1	1
THYM	Thymoma	118	0	0
UCEC	Uterine Corpus Endometrioid Carcinoma	179	0	0
UCS	Uterine Carcinosarcoma	57	0	1
UVM	Uveal Melanoma	79	0	0
